# Clustering analysis of HRCT parameters measured using a texture-based automated system: relationship with clinical outcomes of IPF

**DOI:** 10.1186/s12890-024-03092-9

**Published:** 2024-07-30

**Authors:** Jong-Uk Lee, Jong-Sook Park, Eunjeong Seo, Jin Seol Kim, Hae Ung Lee, Yongjin Chang, Jai Seong Park, Choon-Sik Park

**Affiliations:** 1 Department of Medical Bioscience, Graduate School, Soonchunhyang University, 22, Soonchunhyang-ro, Asan, 31538 Korea; 2 Division of Allergy and Respiratory Medicine, Department of Internal Medicine, Soonchunhyang University Bucheon Hospital, 170 Jomaru-ro, Wonmi-gu, Bucheon-si, Gyeonggi-do 14584 Republic of Korea; 3 Clinical Specialist Coreline Soft, 49 World-Cup Bukro 6-gil, Mapogu, Seoul, 03991 Korea; 4 Department of Radiology, Soonchunhyang University Bucheon Hospital, 170 Jomaru-ro, Bucheon, 14584 Korea

**Keywords:** IPF, HRCT, Texture-based automated system, Clustering analysis, Survival

## Abstract

**Purpose:**

The extent of honeycombing and reticulation predict the clinical prognosis of IPF. Emphysema, consolidation, and ground glass opacity are visible in HRCT scans. To date, there have been few comprehensive studies that have used these parameters. We conducted automated quantitative analysis to identify predictive parameters for clinical outcomes and then grouped the subjects accordingly.

**Methods:**

CT images were obtained while patients held their breath at full inspiration. Parameters were analyzed using an automated lung texture quantification system. Cluster analysis was conducted on 159 IPF patients and clinical profiles were compared between clusters in terms of survival.

**Results:**

Kaplan-Meier analysis revealed that survival rates declined as fibrosis, reticulation, honeycombing, consolidation, and emphysema scores increased. Cox regression analysis revealed that reticulation had the most significant impact on survival rate, followed by honeycombing, consolidation, and emphysema scores. Hierarchical and K-means cluster analyses revealed 3 clusters. Cluster 1 (*n* = 126) with the lowest values for all parameters had the longest survival duration, and relatively-well preserved FVC and DLCO. Cluster 2 (*n* = 15) with high reticulation and consolidation scores had the lowest FVC and DLCO values with a predominance of female, while cluster 3 (*n* = 18) with high honeycombing and emphysema scores predominantly consisted of male smokers. Kaplan-Meier analysis revealed that cluster 2 had the lowest survival rate, followed by cluster 3 and cluster 1.

**Conclusion:**

Automated quantitative CT analysis provides valuable information for predicting clinical outcomes, and clustering based on these parameters may help identify the high-risk group for management.

**Supplementary Information:**

The online version contains supplementary material available at 10.1186/s12890-024-03092-9.

## Introduction

Idiopathic pulmonary fibrosis (IPF) is a progressive fibrotic interstitial pneumonia characterized by extensive deposition of extracellular matrix. It is characterized by scattered subepithelial fibroblast foci composed of proliferating fibroblasts and myofibroblasts, leading to decreased lung volume. These changes are correlated with long-term mortality [1–3]. About half of patients die within 5 years of diagnosis; however, the natural course can show rapid decline, slow progression, or relative stability [4, 5]. Therefore, there is a need for diagnostic tools to predict clinical outcomes. Clinical characteristics associated with survival include age, male gender, current smoking, use of oxygen, and baseline as well as changes in pulmonary function tests (PFTs) [4]. In a previous cluster analysis using clinical parameters identified 4 groups, which differed significantly in the monthly decline of forced vital capacity (FVC) [6]. However, diverse clinical manifestations of IPF have hindered the development of accurate prognostic markers [7]. Therefore, additional approaches have been introduced to improve the prediction.

High-resolution computed tomography (HRCT) of the chest is the standard method for diagnosing idiopathic pulmonary fibrosis (IPF) with positive diagnostic values ranging from 90 to 100% [8, 9]. Furthermore, the manually or automatically measured levels of fibrosis and honeycombing can predict clinical outcomes, including mortality [10–16]. However, the main histopathological feature is a heterogeneous appearance, with areas of fibrosis alternating with less affected or normal parenchyma [17]. Other parameters, including emphysema, consolidation, and ground glass opacity (GGO), have also been studied, but their prognostic associations are controversial [14, 18]. Using fibrosis score (FS) and emphysema index, 3 clusters of IPF have been reported [18]. The prognosis is better in a cluster with less fibrosis and emphysema and a higher FVC compared to the other clusters. However, there have been few clustering studies reported to date that utilized automatic quantitative CT analysis. Therefore, we conducted automated quantitative CT analysis to identify HRCT parameters that can predict clinical outcomes. We also clustered the subjects based on the HRCT parameters in order to identify the high-risk group for mortality.

## Materials and methods subjects

After receiving approval from the Institutional Review Board (IRB) of Soonchunhyang University Bucheon Hospital (IRB No: SCHBC2023-06-014), we retrospectively obtained clinical data and HRCT images of patients with IPF who underwent HRCT between January 2000 and December 2020 (schbc-biobank-2021-008-01). The requirement for informed consent was waived by the IRB of Soonchunhyang University Bucheon Hospital. IPF was diagnosed based on 2011 and 2018 guidelines [8, 19]. They showed no evidence of underlying collagen vascular diseases according to laboratory tests and clinical manifestations. FVC, forced expiratory volume in 1 s (FEV1), and diffusing capacity of the lungs for carbon monoxide (DLCO) were assessed using Vmax 22 (SensorMedics, Yorba Linda, CA, USA) and MasterScreen Body (Jaeger Co., Wurzburg, Germany), and expressed as percentages of predicted values calculated using the Morris and Jones-Meade equations. The subjects were followed up at 3-month intervals. Survival duration was evaluated as the time between the date of diagnosis and date of death or the last follow-up.

### Quantitative measurement of HRCT parameters

CT images were obtained during breath-holding at full inspiration using a 16-channel multi-detect CT scanner (Sensation 16; Siemens, Forchheim, Germany). The HRCT scanning was conducted as follows: 0.75 mm collimation, 1 mm slice thickness, 10 mm interval, sharp kernel (B70f) at 220 mAs with 120–140 kVp, and a matrix size of 512 × 512 pixels. The window settings were as follows: center, − 750 Hounsfield units (HU); width, 1500 HU. Lung texture patterns were analyzed using a texture-based automated quantification system (AVIEW software; Coreline Soft, Seoul, Korea) as described previously [20]. The volumes of the 5 parameters are presented as percentages (referred to as scores) relative to the total lung volume as previously provided [21, 22].

### Cluster and discriminant analyses

An unsupervised clustering analysis was conducted using a two-step approach. In the initial step, the optimal number of clusters was determined using hierarchical cluster analysis and the silhouette method. This was implemented in the NbClust R package, utilizing Ward’s method. In the next step, k-means cluster analysis was conducted using the clusterCirt R package.

### Statistical analysis

Skewed variables were presented as median values with interquartile range (25th and 75th percentiles), while normally distributed variables were presented as mean ± standard error. Kruskal-Wallis, one-way ANOVA, and chi-square tests were employed to compare non-parametric continuous variables, parametric continuous variables, and categorical variables, respectively. For post-hoc analysis, the Mann-Whitney U test was used to compare continuous nonparametric variables between two groups, and Bonferroni’s post hoc test was used for comparisons between two groups of parametric samples. Receiver operating characteristic (ROC) analysis was performed to determine the optimal cut-off value with the highest Youden index. Differences in the areas under the curve (AUC) were compared using Z-tests conducted with MedCalc Statistical Software (v. 12.2.1.0; MedCalc Software, Ostend, Belgium) [23]. Survival rates were compared using Kaplan–Meier survival analysis and the log-rank test. Cox proportional hazard regression models with backward elimination were used to identify independent risk factors for the survival rate. In all analyses, *p* < 0.05 was considered significant.

## Results

### Study subjects

After excluding 11 cases complicated with acute exacerbation, pneumonia, or lung cancer at enrollment, HRCT images of 159 IPF patients were analyzed (Table 1). A total of 63 patients had undergone a surgical lung biopsy, whereas 96 patients were diagnosed after multidisciplinary discussion without lung biopsy. Males predominated and about half of the subjects were ex- or current smokers. The median follow-up duration for the patients was 5.1 years. FVC and FEV1 data were available for all subjects and DLCO data were available for 139 subjects. BAL cell analysis were performed on 113 subjects.

**Table 1:** Clinical characteristics of the study subjects

Parameters	Results
No.	159
Age (year)	71 (41–86)
BMI (kg/m2)	24.1 (22.2–26.3)
Sex (male/female)	94/65
Smoke (NS/ES/CS)	79/51/29
Survival/Death	101/58
Follow up duration (year)	5.1 (1.73–7.78)
FVC (% pred.)	72.3 ± 17.6
DL_CO_ (% pred.) *	66.7 ± 22.9
FEV1/FVC (%)	84.6 ± 7.2
Treatment	
Steroid	104 (65.4)
Immunosuppressants	46 (28.9)
Anti-fibrotic agent	33 (20.8)
BAL total cell count (x10^5^) **	37.3 (3.8–100)
Macrophages (%)	70.6 (43.1–84.7)
Neutrophils (%)	20.8 (7.5–44.8)
Eosinophils (%)	1.4 (0.4–3.4)
Lymphocytes (%)	3.4 (1.2–6.3)

NE/ES/CS, never-smoker/ex-smoker/current-smoker; BMI, body mass index; FVC, forced vital capacity; FEV1, forced expiratory volume in 1 s; DLco, diffusing capacity for carbon monoxide. *:*n* = 139, **:*n* = 114. Data are presented as median values with 25% and 75% quartiles for skewed variables, as means ± SEM for those with normal distributions, or number (%), unless otherwise indicated

### Distribution of HRCT parameters and their associations with survival rates according to Kaplan-Meier and cox proportional hazards analyses

**Fig. 1:**
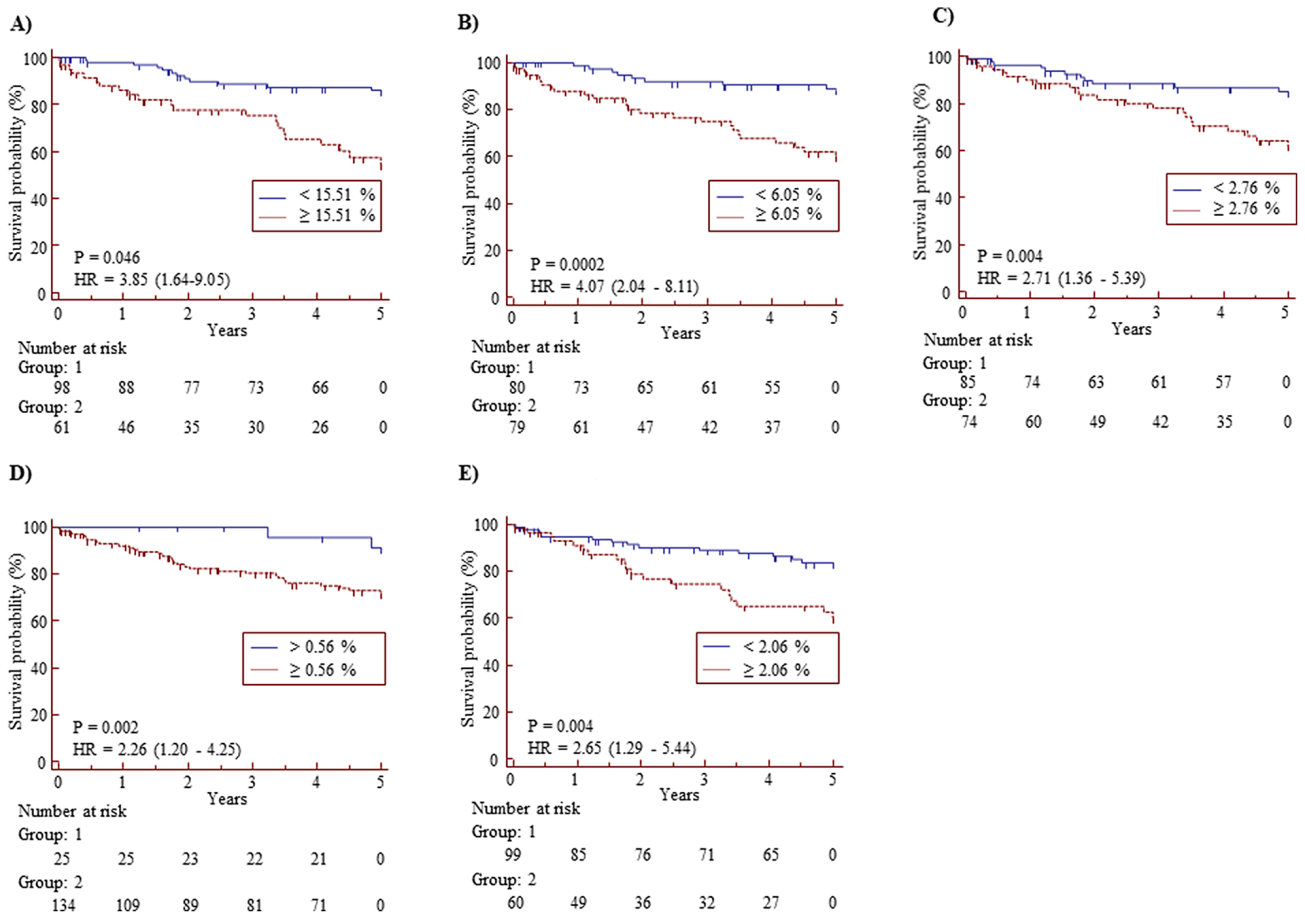
Kaplan-Meier plots of HRCT parameters illustrating survival rates in 159 patients with IPF. Hazard ratios (HRs) with 95% confidence intervals (CIs) and p-values for different cutoffs are presented for the following parameters: (**A**) fibrosis, (**B**) reticulation, (**C**) honeycombing, (**D**) consolidation, and (**E**) emphysema. Data in the boxes indicate cutoff values for each parameter

Reticulation received the highest score, followed by emphysema, honeycombing, consolidation, and GGO (Table 2). The fibrosis score (FS) was calculated by summing the reticulation and honeycombing scores. These parameters were analyzed to predict survival rates in univariate analyses (Table S1). In Kaplan-Meier analyses, a shorter survival rate was associated with higher reticulation as well as honeycombing, consolidation, and emphysema scores (Fig. 1A-E). In the Cox proportional hazards model over the 5-year follow-up period, survival rates were most significantly associated with the reticulation score, followed by the consolidation and honeycombing scores as well as the emphysema score (Table 3).

**Table 2:** Distribution of HRCT parameters of 159 IPF subjects

Variables	Percentages
Normal lung	81.60 (71.05–92.30)
Fibrosis	11.87 (4.92–19.31)
Reticulation	5.79 (3.18–10.59)
Emphysema	1.90 (0.80–3.89)
Honeycomb	1.68 (0.24–7.61)
Consolidation	0.21 (0.09–0.53)
GGO	0.01 (0.00-0.11)

GGO, Ground-glass opacity. Fibrosis score was calculated by summation of reticulation and honeycomb. Data are presented as percentages of the total lung volume and medians with 25–75 percentiles

**Table 3:** Association of HRCT parameters with survival rates on Cox proportional hazards analysis of 159 patients with IPF

Survival duration	Parameter	HR (95% CI)	*p*-value
0 ~ 5 (year)	Fibrosis score	1.070 (1.043–1.098)	< 0.0001
	Reticulation	1.097 (1.06–1.136)	< 0.0001
	Honeycomb	1.044 (1.009–1.081)	0.014
	Consolidation	1.163 (1.024–1.321)	0.02
	Emphysema	1.068 (1.002–1.138)	0.044
	GGO	0.991 (0.866–1.134)	0.894

GGO, Ground-glass opacity; HR, Hazard ratio; CI, confidence interval. Fibrosis score was calculated by summation of reticulation and honeycomb. Results were obtained using Cox multivariate analysis adjusted with the covariates of FVC and DLCO

### Characteristics of the three clusters

**Fig. 2:**
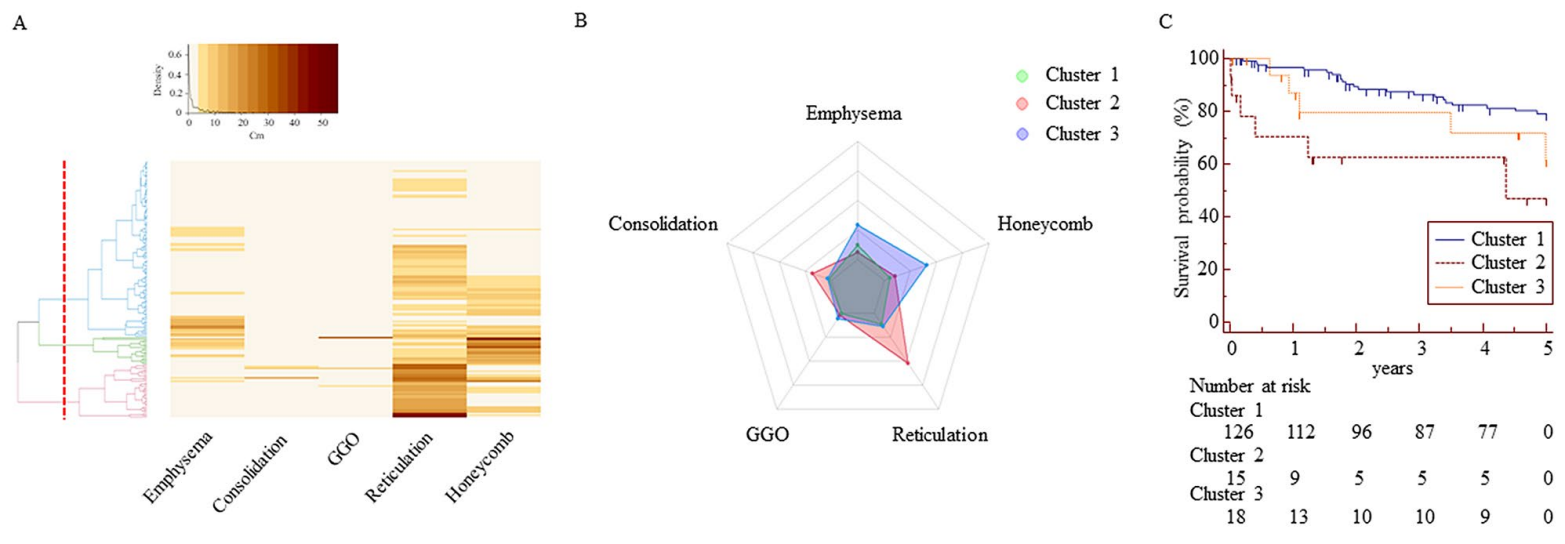
Cluster analysis of HRCT parameters for 159 patients with IPF. (**A**) Heat map and hierarchical clustering. (**B**) Radar plot illustrating the distribution of HRCT parameters across clusters. (**C**) Kaplan-Meier plots of the three clusters illustrating survival rates

Hierarchical and K-means analyses of these HRCT parameters indicated that a three-cluster solution was likely optimal. The heatmap and radar plot are presented in Fig. 2. Cluster 1 (C1) was the largest cluster (*n* = 126), followed by C3 (*n* = 18), and C2 (*n* = 15) (Table 4). The median reticulation and consolidation scores were highest in C2 (28.8% and 1.1%, respectively), while the highest honeycombing and emphysema scores were observed in C3 (18.4% and 6.1%, respectively). These parameters differed significantly between C2 and C3 (*p* < 0.001), while FS did not (32.5% vs. 25.3%, respectively, *p* = 0.055). The scores for parameters, except GGO, were lowest in C1. C2 was predominantly female (60%) and had the lowest FVC and DLCO values, while C3 was predominantly male (83.3%) and comprised current and ex-smokers with relatively preserved FVC. Kaplan-Meier survival analysis adjusted with an anti-fibrotic agent revealed that C2 had the shortest survival time and the lowest survival rate in the cluster group (C1 vs. C2, HR: 3.63, 95% CI: 1.46–8.97, *p* < 0.001; C1 vs. C3, HR: 1.41, 95% CI: 0.87–2.29, *p* = 0.163; C2 vs. C3, HR: 1.67, 95% CI: 0.5–5.5, *p* = 0.396) (Fig. 2).

**Table 4:** Comparison of clinical characteristics between 3 clusters

Variables	Cluster 1 (*n* = 126)	Cluster 2 (*n* = 15)	Cluster 3 (*n* = 18)	*p*-value
Normal lung (%)	81.5 (81.5–93.8)	64.9 (52.1–69.6) *	68.6 (59.6–72.7) *	0.885
Emphysema (%)	0.7 (0.7–2.8)	0.8 (0.5–1.7) †	6.1 (3.1–9.3) *	< 0.001
Consolidation (%)	0.1 (0.1–0.5)	1.1 (0.4–2.9) *†	0.3 (0.1–0.5)	< 0.001
GGO (%)	0 (0–0)	0 (0-0.1)	0 (0–0)	0.824
Reticulation (%)	2.8 (2.8–9.8)	28.8 (21.6–30.6) *†	6.6 (4.3–9.3)	< 0.001
Honeycomb (%)	0.4 (0.3–5.3)	4.1 (1.8–7.9) *†	18.4 (14.9–23.2) *	< 0.001
Fibrosis score (%)	4.3 (4.3–15.1)	32.5 (28.1–43.2) *	25.3 (22.9–29.7) *	< 0.001
Age (year)	70.6 (64.5–77.0)	71.7 (66.8–79.7)	71.2 (68.9–79.0)	0.259
BMI (kg/m2)	22.2 (22.2–26.3)	23.7 (22.4–26.8)	23.3 (22.5–25.4)	0.901
Male (%)	58.7	40 †	83.3	0.036
Smoke (NS/ES/CS)	66/31/26	9/5/1 †	2/14/2 *	0.001
Survival/Death	84/42	7/8	10/8	0.238
Survival duration (years)	5.9 (2.2–8.4)	1.3 (0.1–3.7) *	4.0 (0.9–5.5) *	< 0.001
FVC (% pred.)	74.5 ± 17.6	53.4 ± 13.4 *	67.9 ± 14.4	0.0052
DL_CO_ (% pred.)	71.0 ± 21.9	41.8 ± 20.2 *	49.1 ± 14.9 *	< 0.001
Treatment				
Steroid	80 (63.5)	12 (80)	12 (66.7)	0.443
Immunosuppressants	32 (25.4)	7 (46.7)	8 (44.4)	0.157
Anti-fibrotic agent	29 (23)	1 (6.7)	3 (16.7)	0.303

GGO, Ground-glass opacity; NE/ES/CS, never-smoker/ex-smoker/current-smoker. BMI, body mass index; FVC, forced vital capacity; DLco, diffusing capacity for carbon monoxide. Fibrosis score was calculated by summation of reticulation and honeycomb. Data are presented as median values with 25% and 75% quartiles for skewed variables, as means ± SEM for those with normal distributions, or number (%), unless otherwise indicated. *: *P* < 0.05 compared with Cluster 1, †: *P* < 0.05 compared with Cluster 3

## Discussion

In the present study, the prognosis was worst for C2, which had the highest reticulation and consolidation scores, followed by C3, which had the highest honeycombing and emphysema scores. The survival time of C2 was one-third shorter than that of C3. These two clusters accounted for 20% of the total study population, with C2 representing 9% and C3 representing 11%. The remaining 80% of the subjects (C1) experienced longer survival times and higher survival rates. C2 was predominantly composed of non-smokers and female subjects, while C3 was predominantly composed of current and ex-smokers and male subjects. When analyzed in the entire study population, the mortality risk was similar between males and females (*p* = 0.78) (Figure S1). Based on this data, we have demonstrated, to the best of our knowledge, for the first time, that the cluster primarily consisting of non-smoking females may have the most severe course when they exhibit extensive reticulation and consolidation on HRCT.

In general, IPF affects males more frequently than females, and environmental risk factors such as smoking are commonly associated with the development of IPF [24]. A recent nationwide study, using Korean insurance claims data, reported a 1.6 times higher prevalence of IPF in males than in females [25, 26]. In the present study, the male-to-female ratio was 1.44, and exposure histories to risk factors were not available, except for smoking. Interestingly, all of the female subjects in C2 were never-smokers (Table S2). Therefore, smoking is not a risk factor for a poor prognosis in this group.

Although male sex is a risk factor for increased mortality in ILD [27–29], substantial heterogeneity is present between studies [30]. In the present study, survival rates were similar between the sexes when analyzed in the whole study subjects (Figure S1. Recently, Assayag et al. reported that mortality was higher in female than male patients with IPF (HR = 2.21) maybe due to underdiagnoses in comparison with males [31]. In the present study, the survival time of C2 was shorter than that of C3, particularly among female subjects (Table S1). These data suggest the possibility of the delayed diagnosis and treatment of IPF in females at the time of enrollment. Staging systems for IPF have been based on a combination of composite physiological indexes, such as GAP index [28]. In the present study, C2 exhibited the lowest FVC and DLCO values, while C3 showed relatively preserved FVC. Therefore, the disruption of lung volume may be a factor contributing to the short survival time of C2. It has been revealed that adding fibrotic scores from HRCT provides better prediction compared to using the GAP index alone [32]. Cox analysis using GAP index was not conducted in the present study because female subjects in C2 had the worst prognosis. However, when adjusted for the covariates of FVC, DLCO and age as components of the Cox index, both reticulation and honeycombing showed an increased hazard ratio for survival rate (Table S3). Our findings, indicating that reticulation and honeycombing scores are the most reliable metrics for predicting survival, align with a prior study that employed automated assessments of 144 IPF patients [33]. However, the fibrosis, reticulation, and honeycombing scores in our study were approximately half of those in that study (22.8%, 15.9%, and 6.8%, respectively). The discrepancies between the two studies may be due to differences in IPF stages; lung functions were relatively preserved in the present study compared to those of that study (FVC: 73% vs. 70% and DLCO: 66% vs. 46%, respectively).

Different survival rates have been reported for emphysema patients with IPF, including rates that are worse, similar, and better than those for IPF alone [34–36]. Combined pulmonary fibrosis and emphysema (CPFE) has been recognized as a distinct medical condition [14, 33, 35, 37, 38]. In a study of 365 patients with IPF [39], coexisting pulmonary fibrosis with 10% or more emphysema was observed in 8% of the patients, and the survival rate was similar between those with and without CPFE. In the current study, 8.18% of subjects had ≥ 10% emphysema (*n* = 13/159), and no difference in survival was observed between the two groups divided by an emphysema cutoff of threshold (data not shown). Recently, Bak et al. have demonstrated that the FS and emphysema index could be used to distinguish three clusters of IPF [40]. The prognosis is better in the cluster characterized by less fibrosis and emphysema, and high FVC and GGO scores, than in the cluster with higher FS and emphysema scores. In our study, emphysema score was higher in C3 than C2 suggesting that a high emphysema score may be a favorable prognostic marker in subjects with high FS. Emphysema with a thickened wall may be interpreted as honeycombing [41]. In the present study, the emphysema score exhibited a significant correlation with the honeycombing score (*r* = 0.438, *p* < 0.001), but not with the reticulation score (*r* = −0.145, *p* = 0.068, Table S4). A comparative study revealed that visual analysis is superior to automatically measured score to quantify emphysema, possibly because automated readings of destructive emphysema are confounded by honeycombing [42]. Accordingly, the extent of emphysema should be interpreted differently as a prognostic parameter, taking into account FS and visual assessment may be necessary in quantitative analysis of emphysema surrounding reticulation. In the present study, the consolidation (0.29%) and GGO (0.01%) scores were lower compared to the other parameters. However, higher consolidation scores were linked to lower survival rates. It is widely accepted that consolidation and nodules are uncommon radiological findings in cases of IPF without complications [43, 44]. Therefore, we excluded subjects with acute exacerbations, acute or chronic infections, and incidental lung cancer at enrollment.

This study had some limitations. First, there may have been bias due to the retrospective and cross-sectional design of the single-center study. Secondly, in the present study, we utilized a 16-row Siemens CT scanner with a 1.0 mm thickness and 10.0 mm interval, which led to gaps between slices and partial volume artifacts. These artifacts may have compromised the accuracy of imaging quantification in our study on IPF, potentially influencing the study’s findings. Additionally, the algorithm used in the study for HRCT quantification cannot differentiate between bronchiectasis and traction. Although validation was not conducted, areas with bronchiectasis are mostly categorized as honeycombing or reticular patterns. Therefore, quantification of reticular opacities or honeycombs may include assessing traction bronchiectasis. Thirdly, the number of patients in C2 and C3 was small. Therefore, more patients in advanced stages will be recruited for consensus clustering. Fourthly, the survival rate was not adjusted for confounding factors such as lung cancer and pulmonary hypertension, and other comorbidities that could affect the survival of patients with IPF. Finally, HRCT parameters were not assessed for their associations with other clinical outcomes, such as disease progression or acute exacerbations. Therefore, additional prospective and longitudinal long-term assessments are necessary to confirm our findings in a larger cohort.

## Conclusions

Quantitative analysis of HRCT parameters provides valuable information for predicting clinical outcomes. Emphysema and consolidation scores, as well as reticulation and honeycombing, are linked to a decreased survival rate in IPF. Among the 3 clusters identified, the most common cluster exhibiting the lowest values for all five HRCT parameters indicates the best prognosis. The cluster with the highest reticulation and consolidation scores has the worst prognosis, followed by the cluster with the highest honeycombing and emphysema scores. Consequently, the clustering of HRCT parameters measured through automated quantitative analysis techniques may prove beneficial in identifying the high-risk group of individuals with IPF. Automated quantitative CT analysis provides valuable information for predicting clinical outcomes, and clustering based on these parameters may help identify the high-risk group for management.

### Electronic supplementary material

Below is the link to the electronic supplementary material.

Supplementary Material 1

Supplementary Material 2

## Data Availability

The datasets used and/or analysed during the current study available from the corresponding author on reasonable request.
