# Supplementary material for: Clustering analysis of HRCT parameters measured using a texture-based automated system: relationship with clinical outcomes of IPF
Source: BMC Pulm Med. 2024 Jul 30;24:367. doi: 10.1186/s12890-024-03092-9 (PMC11290077; doi:10.1186/s12890-024-03092-9)
Supplement: Supplementary file 1 — Supplementary Material 1 [file 12890_2024_3092_MOESM1_ESM.docx]

Table S1. Association of HRCT parameters with survival rates of 159 subjects using Kaplan-Meier analysis.

| Parameters | ROC curve | | | Kaplan-Meier survival analysis | |
| --- | --- | --- | --- | --- | --- |
|  | AUC | p-value | Cut-off value (%) | HR (95% CI) | p-value |
| Fibrosis score | 0.622 | 0.009 | 15.51 | 3.15 (1.76-5.64) | <0.001 |
| Reticulation | 0.602 | 0.032 | 6.05 | 2.85 (1.68 - 4.82) | <0.001 |
| Honeycomb | 0.603 | 0.025 | 2.76 | 2.31 (1.36 - 3.90) | 0.001 |
| Consolidation | 0.57 | 0.141 | 0.56 | 2.26 (1.20 - 4.25) | 0.002 |
| Emphysema | 0.533 | 0.818 | 2.06 | 1.73 (1.00 - 2.98) | 0.034 |
| GGO | 0.517 | 0.731 | 0.03 | 0.76 (0.44 - 1.30) | 0.344 |

ROC, receiver operating characteristic; HR, Hazard ratio; CI, confidence interval; GGO, Ground-glass opacity. The fibrosis score was calculated by summing the reticulation and honeycomb. Results were obtained through univariate analysis.

Table S2. Comparison of clinical characteristics between 3 clusters by sex

| Parameters | Cluster 1 (n=126) | | | Cluster 2 (n=15) | | | Cluster 3 (n=18) | | |
| --- | --- | --- | --- | --- | --- | --- | --- | --- | --- |
|  | Male (n=74) | Female (n=52) | p-value | Male (n=6) | Female (n=9) | p-value | Male (n=15) | Female (n=3) | p-value |
| FVC (% pred.) | 73.8±18.1 | 72.1±17.4 | 0.650 | 57.6±7.6 | 58.9±18.2 | 0.514 | 70.43±12.5 | 72.25±4.3 | 0.650 |
| DL_CO_ (% pred.) | 73.8±21.8 | 63.4±21.2 | 0.014 | 55.3±24.1 | 45.5±20.1 | 0.382 | 47.69±13.8 | 57.33±19.9 | 0.014 |
| Follow up duration (year) | 5.9 (2.1-8.9) | 5.9 (2.0-7.7) | 0.783 | 2.8 (0.3-4.3) | 1.2 (0.1-2.6) | 0.050 | 2.2 (0.9-5.2) | 6.7 (4.4-10.3) | 0.783 |
| Hazard ratio with  95% confidence intervals | - | 0.675 (0.288-1.579) | 0.387 | - | 1.348 (0.263-6.891) | 0.727 | - | 0.560 (0.065-4.790) | 0.560 |
| Smoke (NS/ES/CS) | 21/29/24 | 47/3/2 | <0.001 | 0/5/1 | 9/0/0 | 0.003 | 0/13/2 | 2/1/0 | <0.001 |

FVC, forced vital capacity; DLco, diffusing capacity for carbon monoxide; NE/ES/CS, never-smoker/ex-smoker/current-smoker.

Table S3. Association of HRCT parameters with survival rates using Cox proportional hazards analysis, with covariates including FVC, DLCO and age.

| Follow-up duration | Parameter | HR (95% CI) | p-value |
| --- | --- | --- | --- |
| 0~5 (year)  (n=159) | GGO | 0.999 (0.942-1.059) | 0.965 |
|  | Emphysema | 1.031 (0.965-1.101) | 0.366 |
|  | Consolidation | 1.124 (0.97-1.303) | 0.121 |
|  | Honeycomb | 1.03 (0.998-1.063) | 0.071 |
|  | Reticulation | 1.074 (1.032-1.118) | <0.001 |
|  | Fibrosis score | 1.046 (1.02-1.072) | <0.001 |

GGO, Ground-glass opacity; HR, Hazard ratio; CI, confidence interval. The fibrosis score was calculated by summing the reticulation and honeycomb.

Table S4. Correlation among the scores of HRCT parameters

| Parameters |  | Emphysema | Consolidation | GGO | Reticulation | Honeycomb | Fibrosis score |
| --- | --- | --- | --- | --- | --- | --- | --- |
| Emphysema | r | 1 | -0.265 | 0.007 | -0.145 | 0.438 | 0.126 |
|  | p-value | - | <0.001 | 0.931 | 0.068 | <0.001 | 0.115 |
| Consolidation | r |  |  | 0.144 | 0.525 | 0.156 | 0.444 |
|  | p-value |  |  | 0.071 | <0.001 | 0.049 | <0.001 |
| GGO | r |  |  |  | 0.235 | 0.02 | 0.173 |
|  | p-value |  |  |  | 0.003 | 0.802 | 0.029 |
| Reticular  opacity | r |  |  |  |  | 0.322 | 0.827 |
|  | p-value |  |  |  |  | <0.001 | <0.001 |
| Honeycomb | r |  |  |  |  |  | 0.725 |
|  | p-value |  |  |  |  |  | <0.001 |
| Fibrosis score | r |  |  |  |  |  | 1 |
|  | p-value |  |  |  |  |  | - |

GGO, Ground-glass opacity. Fibrosis score was calculated by summation of reticulation and honeycomb. Correlation coefficient was calculated using Spearman's rank correlation.
