# Supplementary material for: Clustering analysis of HRCT parameters measured using a texture-based automated system: relationship with clinical outcomes of IPF
Source: BMC Pulm Med. 2024 Jul 30;24:367. doi: 10.1186/s12890-024-03092-9 (PMC11290077; doi:10.1186/s12890-024-03092-9)

**Figure S. Kaplan-Meier plots illustrating the survival rates of 95 male and 64 female patients with IPF.** Hazard ratios with 95% confidence intervals (CIs) and p-values are provided.


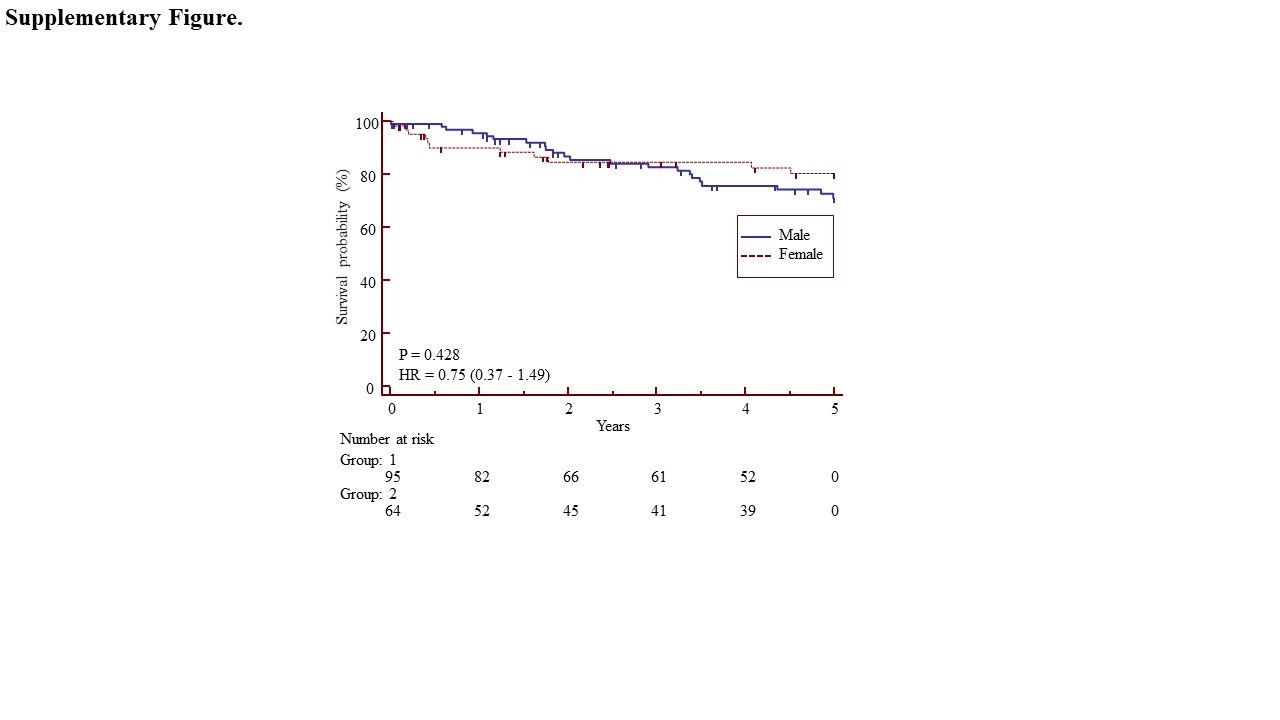

Supplement: Supplementary file 2 — Supplementary Material 2 [file 12890_2024_3092_MOESM2_ESM.docx]
